# *Znf703* is a novel RA target in the neural plate border

**DOI:** 10.1038/s41598-019-44722-1

**Published:** 2019-06-04

**Authors:** Amanda Janesick, Weiyi Tang, Kristen Ampig, Bruce Blumberg

**Affiliations:** 1 Department of Developmental and Cell Biology, 2011 Biological Sciences 3, University of California, California, Irvine 92697-2300 USA; 2 Department of Pharmaceutical Sciences, University of California, California, Irvine USA; 3 Present Address: Department of Otolaryngology–Head & Neck Surgery, Stanford University School of Medicine, Stanford, CA 94305 USA; 4 Present Address: Division of Biology, California Institute of Technology, Pasadena, CA USA

**Keywords:** Embryology, Xenopus, Pattern formation

## Abstract

*Znf703* is an RAR- and Wnt-inducible transcription factor that exhibits a complex expression pattern in the developing embryo: *Znf703* mRNA is found in the early circumblastoporal ring, then later throughout the neural plate and its border, and subsequently in the mid/hindbrain and somites. We show that *Znf703* has a different and separable function in early mesoderm versus neural crest and placode development. Independent of its early knockdown phenotype on *Gdf3* and *Wnt8*, *Znf703* disrupts patterning of distinct neural crest migratory streams normally delineated by *Sox10, Twist*, and *Foxd3* and inhibits otocyst formation and otic expression of *Sox10* and *Eya1*. Furthermore, *Znf703* promotes massive overgrowth of SOX2+ cells, disrupting the SoxB1 balance at the neural plate border. Despite prominent expression in other neural plate border-derived cranial and sensory domains, *Znf703* is selectively absent from the otocyst, suggesting that *Znf703* must be specifically cleared or down-regulated for proper otic development. We show that mutation of the putative Groucho-repression domain does not ameliorate *Znf703* effects on mesoderm, neural crest, and placodes. We instead provide evidence that *Znf703* requires the Buttonhead domain for transcriptional repression.

## Introduction

The neural plate border and non-neural ectoderm comprise a narrow arc of multipotent cells that circumscribe the neural plate^1^. Although cells of different lineages are comingled in this region early, they eventually segregate into neural, placodal, neural crest, and epidermal fates^1–3^. *Znf703* belongs to a highly conserved family of NET (*Noc*, *Nlz*, *Elbow*, and *Tlp-1*) zinc finger transcriptional repressors and was previously studied in zebrafish, primarily in the mid/hindbrain^4,5^. *Znf703* was recently shown to be expressed in the neural plate border of *Xenopus* where it partially overlaps with presumptive neural crest (*Snai2*)^6^. We recently identified *Znf703* as a target of RARγ^7^, prompting further investigation into the role of *Znf703* in early developmental processes that are sensitive to retinoic acid, such as neural crest and placodal patterning.

Because it is expressed at the neural plate border, *Znf703* protein is positioned to interact with a variety of signaling pathways. *Znf703* inhibits *Wnt* and *Tgfβ* signaling *in vitro*^8,9^, and reciprocally, *Znf703* is a direct target of *Wnt/β-catenin* signaling in 293 T human embryonic kidney (HEK) cells, mouse, and *Xenopus* embryos^8,10,11^. There is also evidence that *Znf703* is a direct target of *T* (*Brachyury*) in both mouse and *Xenopus*^12,13^. Furthermore, *Znf703* is induced by retinoic acid (RA) signaling in early and late zebrafish gastrula embryos^14^. We recently showed that activation of RARγ1 is required for *Znf703* expression at gastrula stage^7^.

Here we show that *Znf703* is strongly modulated by RA throughout development: *Znf703* mRNA is vastly expanded dorsally and rostrally by RA, obliterating the mid-hindbrain and neural plate border boundaries. We further show that mis/overexpression of *Znf703* blurs the *Sox10* expression domain such that normally segregated migratory streams (branchial, hyoid and mandibular) of neural crest are collapsed, aggregated, and devoid of overt patterning. We found that mis/overexpression of *Znf703* causes a massive expansion of SOX2+ cells, while inhibiting expression of the placode marker, *Eya1* as well as otic *Sox10* expression. This results in the shrinkage or disappearance of the hollow ball of ectoderm that delineates an otocyst. Towards *Znf703* function, we investigated the highly conserved FKPY domain of *Znf703* and found that mutation of FKPY had minimal effect on transcriptional repression and developmental phenotypes compared to wild type, but that the buttonhead domain was indispensable for repression by *Znf703*. Finally, we show that phenotypes on otic and neural crest development are separable from early loss of mesodermal markers *Gdf3* and *Wnt8*. Hence, despite early phenotypes that could potentially affect neural crest competence, *Znf703* is still able to influence neural crest and placode patterning when overexpressed after gastrulation.

## Methods

### Phylogenetic tree

The Noc-family phylogenetic tree was constructed by aligning sequences with the MAFFT v7.306b (E-INS-i algorithm)^15^, then creating the tree using default settings, with bootstrap resampling set to 1000^16^. The resultant tree was drawn with FigTree v1.4.2, rooting at the midpoint^17^.

### Embryo microinjection, treatment, and *in situ* hybridization

All experiments in *Xenopus* were performed in accordance with the relevant guidelines and regulations, and approved by the Institutional Animal Care and Use Committee of the University of California, Irvine. *Xenopus* eggs were fertilized *in vitro* and embryos staged as described^18^. Embryos were microinjected bilaterally or unilaterally at the two- or four-cell stage with *Znf703* (WT, mutant or inducible) mRNA together with 100 pg/embryo *β-galactosidase* (*β-gal*) mRNA lineage tracer (LT). Embryos were maintained in 0.1x MBS until appropriate stages. Embryos processed for whole-mount *in situ* hybridization (WISH) were fixed in MEMFA, stained with magenta-GAL (Biosynth), and then stored in 100% EtOH^18^. We reverse transcribed (Life Technologies) and sequenced the mRNA we microinjected to verify the identity of each *Znf703* mRNA microinjected. For chemical treatments (DEX or TTNPB), embryos were transferred in groups of 25 to 60-mm glass Petri dishes with 10 mL of 0.1X MBS containing chemicals at the following concentrations: 5 μM dexamethasone or corresponding vehicle control (0.05% DMSO); 1 μM TTNPB or corresponding vehicle control (0.1% EtOH).

Whole mount *in situ* hybridization was performed on microinjected embryos as previously described^18^. All probes were prepared via PCR amplification of protein coding regions (~500–800 bp) from either cDNA or library clones with a bacteriophage T7 promoter at the 3′ end. Relevant primers are listed in Table S1. Probes were transcribed with MEGAscript® T7 (Life Technologies) in the presence of digoxigenin-11-UTP (Roche). Double WISH was conducted as previously described^18^. RT-QPCR was conducted as previously described^19^, and relevant primers are listed in Table S2.

### Immunohistochemistry on vibratome sections and whole mounts

Embryos were embedded, sectioned and stained and embedded as described^7^. Transverse sections were labeled with primary antibody anti-SOX2 E-4 (1:100; Santa Cruz Biotechnology) followed by secondary antibody anti-mouse-647 (1:200; ThermoFisher) and DAPI nuclear stain (1:2000). Whole mount preparations were stained with DAPI, followed by clearing and mounting in Scale A2^20^. Embryos were imaged on the Zeiss LSM880 confocal microscope at 20X magnification as described previously^7^.

### Transient transfection and luciferase assays

pCDG1-*Znf703* was constructed by PCR amplification of the protein-coding regions of *Xenopus laevis* cDNA and cloned into the NcoI-BamHI site of pCDG1. pCDG1-*Znf703* mutant constructs were made by two-fragment PCR (primers listed in Table S3) to generate the conservative FKPY→LQAF^21^, or non-conservative FKPY→AAAA substitutions. pCDG1-*Znf703* clones were sequence verified, and linearized with *NotI*. 5′-capped mRNA was transcribed using T7 mMESSAGE mMACHINE® Kit (Thermo Fisher Scientific). pCMX-Gal-*Znf703* constructs were constructed by PCR amplification of pCDG1-*Znf703* and cloned into the EcoRI-BamHI site of pCMX-Gal4^22^ (primers listed in Table S4). COS7 cells were transiently transfected with Gal constructs as previously described^23^. Data are reported as normalized luciferase ± S.E.M. where reduction of luciferase activity compared to Gal4 alone indicates transcriptional repression. Statistical significance was determined using one-way ANOVA and Bonferroni post-hoc test in GraphPad Prism v5.0.

## Results

### Znf703 is a highly conserved transcriptional repressor that exhibits a complex expression pattern in mesoderm, neural plate, and mid/hindbrain

**Figure 1:**
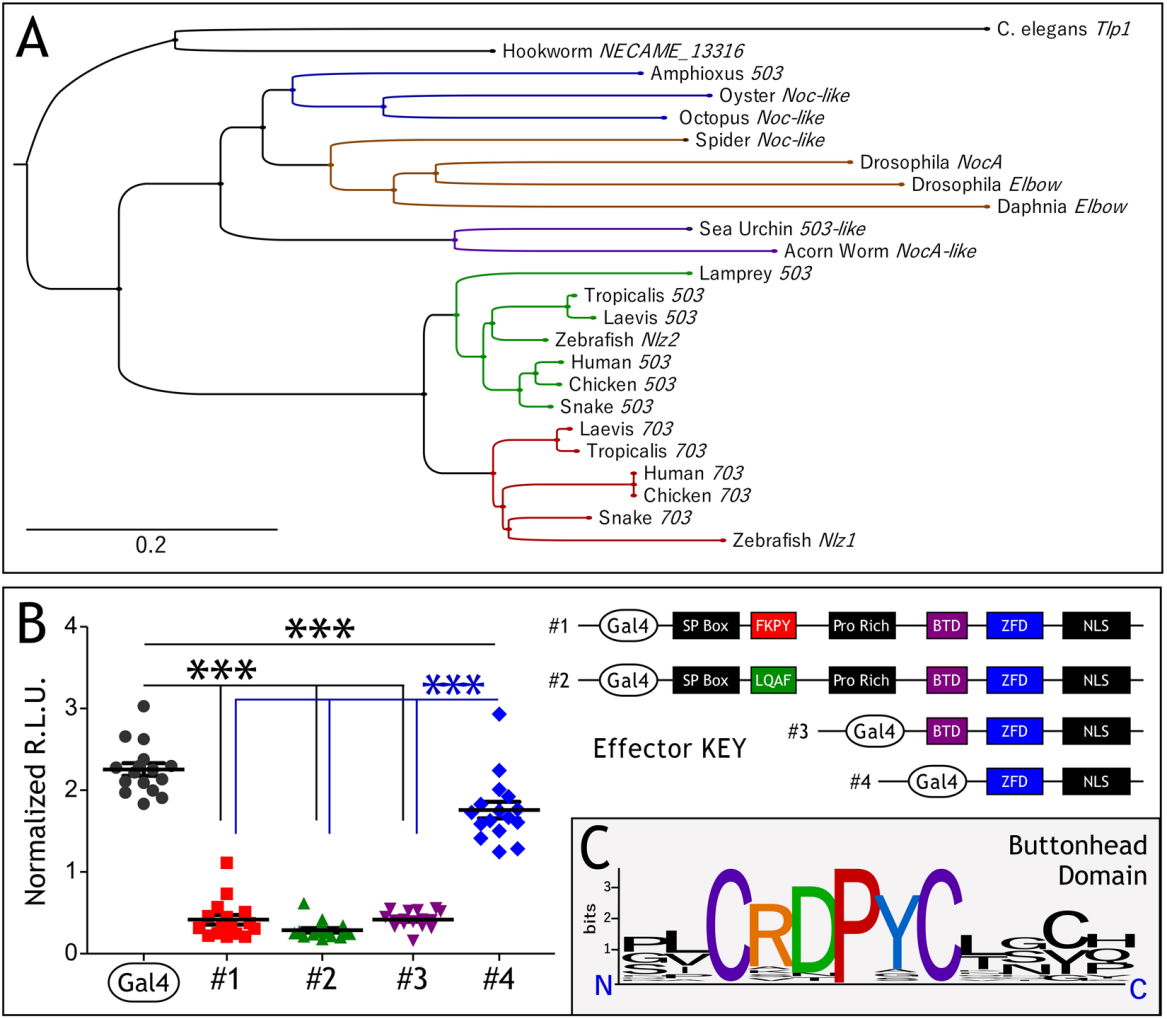
*Znf703* is a conserved transcriptional repressor. (**A**) Phylogenetic tree of *Znf703* sequences aligned and constructed with MAFFT (see materials and methods). The scale bar represents the divergence distance of 0.2 amino acid substitutions per site of the *Znf703* sequence. The tree segregates organisms appropriately with the exception of Amphioxus. We attempted to add more sequences to improve this unexpected result, but Amphioxus persistently segregated with mollusk sequences. (**B**) Cos7 cells were transfected with 5:5:1 DNA ratio of reporter (Gal4-Luc): *β-gal*: effector (*Znf703*). The y-axes represent relative light units measured by the luminometer normalized to *β-gal* activity. Basal reporter activity (Gal4 alone) is repressed by *Znf703*. Mutating the FKPY domain or deletion of the N-terminus up until the *Buttonhead* (*Btd*) domain still represses transcriptional activity. Deletion beyond the *Btd* domain relieves repression. Statistical significance was determined using one-way ANOVA, and Bonferroni post-hoc test in GraphPad Prism v5.0 (***P ≤ 0.001). (**C**) Conservation of the *Btd* domain across the animal kingdom, visualized with WebLogo^66^.

*Znf703* and *Znf503* are members of the NET (Noc, Nlz, Elbow, and Tlp-1) family of zinc finger transcriptional repressors. We constructed a phylogenic tree of representative members of the NET family and show that orthologs of *Znf703* and *Znf503* occur in a diverse number of taxonomic groups (Fig. 1A). ZNF703 and ZNF503 proteins have a conserved FKPY motif which was predicted to recruit Groucho based on its similarity to Brinker and Elbow FKPY^24,25^ and Hucklebein FRPW^26^. We performed transient transfection assays using four different Gal4 effector constructs, mutating the putative Groucho-interacting domain of Xenopus *Znf703* from FKPY to LQAF, or serially deleting the proline rich and buttonhead domains (Fig. 1B). We assayed the ability of these constructs to induce luminescence using a Gal4-luciferase reporter. Reduction of luciferase activity compared to Gal4 alone indicates transcriptional repression by ZNF703. Only deletion past the buttonhead domain relieved repression by ZNF703 (Fig. 1B). The buttonhead domain is highly conserved across all species shown in Fig. 1A, with the two cysteine and proline residues never deviating from the consensus (Fig. 1C). Although neither function nor interacting partners have been characterized for the buttonhead domain, we infer that this domain is likely to be required for transcriptional repression by ZNF703.

**Figure 2:**
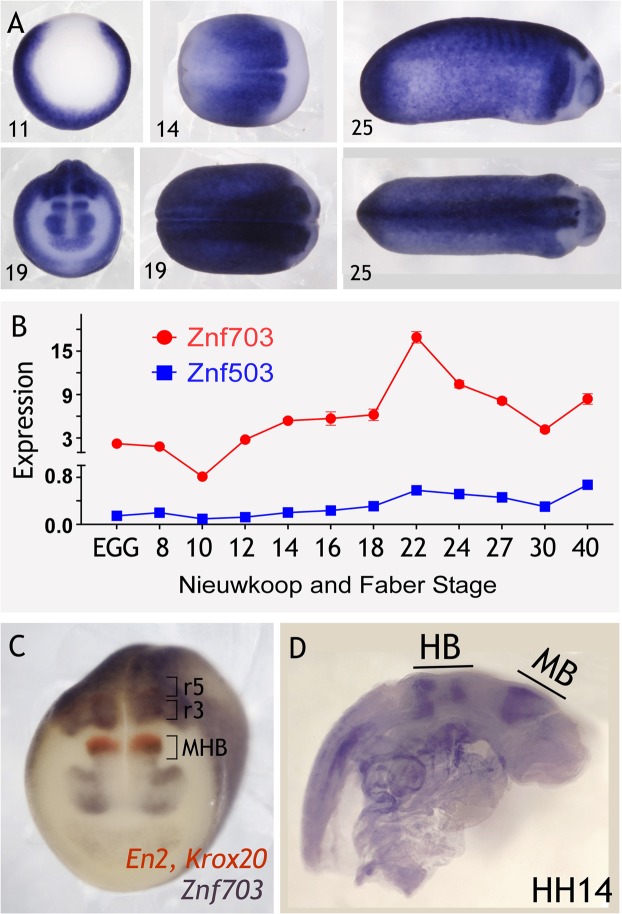
Expression of *Znf703* and *Znf503* across developmental time. (**A**) Whole mount *in situ* hybridization of *Znf703* expression at Nieuwkoop and Faber developmental stages 11 (vegetal view, dorsal at the top), 14 (dorsal view, anterior on the left), 19 (dorsal and anterior views), and 25 (lateral and dorsal views, anterior on the right). WISH of *Znf503* expression can be found in Fig. S1. (**B**) Double WISH at stage 19 reveals the spatial relationship between *Znf703* and midbrain (*Engrailed 2*) and hindbrain (*Krox20*) markers. (**C**) *Znf703* expression in a chick embryo at Hamburger Hamilton (HH) Stage 16 (lateral view; anterior on the right). MB = midbrain; HB = hindbrain. (**D**) QPCR showing *Znf703* and *Znf503* gene expression averaging two biological replicates over developmental time. Error bars = S.E.M. The y-axis represents 2^−ΔCt^ values (adjusted for primer efficiency), normalized to reference gene, *Histone H4*.

We characterized *Xenopus* expression of *Znf703* (Fig. 2A–C) and *Znf503* (Fig. 2B, S1) expression over developmental time. *Znf703* and *Znf503* are expressed in the circumblastoporal ring of the stage 10 gastrula, but absent from the organizer, reminiscent of *Ventx2* or *Wnt8* expression domains. At stage 14, *Znf703* is expressed broadly in both the neural plate and the border zone, but is absent from the anterior. By stage 19, *Znf703* is expressed in the eye/forebrain, absent from r1/r2, then forms a sharp boundary at r3/r4, as shown previously^6^. We also performed WISH for *Znf503* expression, which is weaker and less sharp, but generally shows a similar expression pattern to *Znf703*, albeit without anterior expression at neurula stage (Fig. S1). Xenbase expression data is not available for *X. laevis Znf503*, but *X. tropicalis* shows a 10-to-17-fold magnitude difference in transcript counts between *Znf703* and the lower expressed *Znf503* at stages 11–12.5. Our QPCR expression analysis in *X. laevis* verifies that *Znf703* is more highly expressed than *Znf503* (Fig. 2B).

Double WISH revealed that *Znf703* expression overlaps with *En2* and *Krox2* (Fig. 2C). This mid-hindbrain expression is concordant with zebrafish expression data where *Nlz1* is found at both the mid-hindbrain boundary, and the hindbrain, but only as rostral as rhombomere 3, and later expanding to rhombomere 2^5,14,27^. In chick embryos, *Znf703* also yields sharp boundaries of expression in the hindbrain at E2.5 (~HH14) (Fig. 2D). The murine ortholog, *Zfp703*, also shows similar expression to chicken^8^. These results demonstrate that *Znf703* sequence and spatial expression are highly conserved among fish, birds, amphibians, and mammals.

### Znf703 is modulated by RA

**Figure 3:**
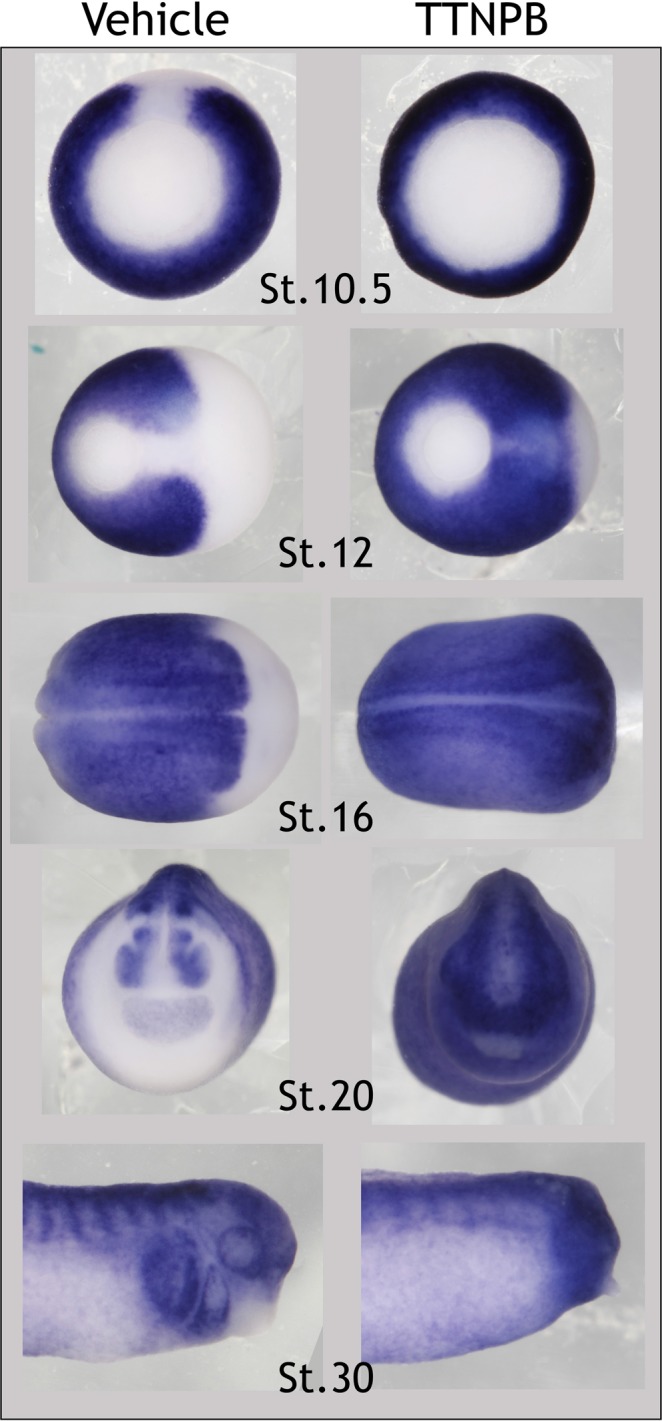
*Znf703* expression is modulated by RAR-selective agonist TTNPB. WISH from embryos treated at stage 6/7 with 1 µM TTNPB or control vehicle (0.1% EtOH). TTNPB greatly expands expression of *Znf703* into the dorsal and anterior domains, relative to control vehicle. Stage 11 embryos are shown in vegetal view with the dorsal lip at the top. Stage 12 and 16 are shown in dorsal view with anterior to the right. Stage 20 embryos are shown in anterior view. Stage 30 embryos are shown in lateral view with anterior to the right.

In Janesick, Tang *et al*., 2018, we identified *Znf703* as a target of RAR signaling by RNA-seq and showed that RARγ1 was required for its expression in the *Xenopus* gastrula. Treatment at stage 6/7 with 1 µM of the RAR-selective agonist, TTNPB, showed that *Znf703* is responsive to modulation of RAR signaling across developmental time (Fig. 3). *Znf703* is normally absent from the organizer, but is ectopically expanded in the presence of TTNPB. By stage 16, TTNPB causes *Znf703* to be expressed throughout the embryo such that the normal anterior boundary of *Znf703* is obliterated–this continues throughout stages 20 and 30. Treatment with the RAR-selective antagonist, AGN193109, elicited only subtle alterations in *Znf703* expression (data not shown).

### Znf703 effect on early mesoderm is separable from its disruption of placode and neural crest patterning

**Figure 4:**
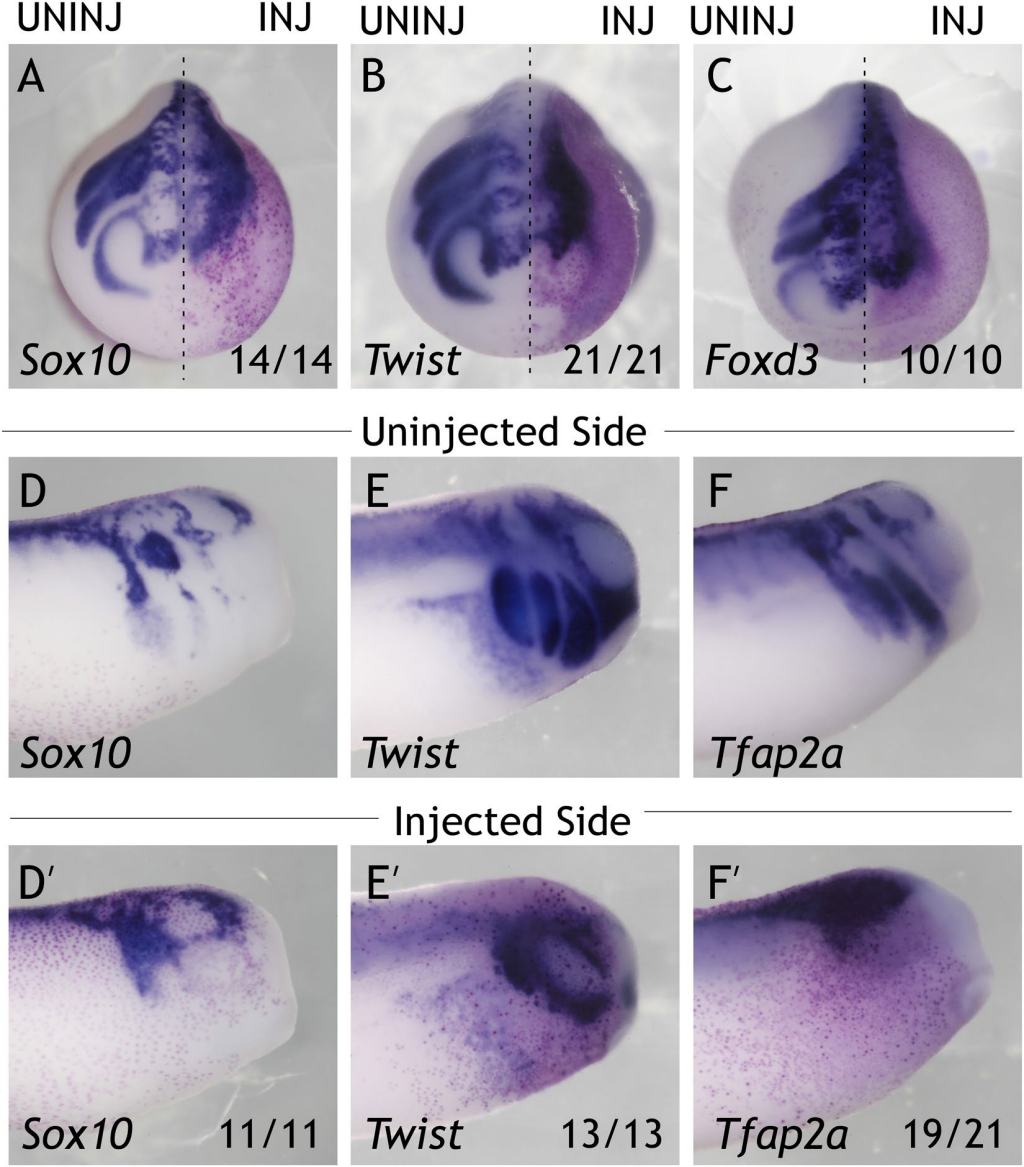
Overexpression of *Znf703* mRNA inhibits neural crest migration. Embryos were injected unilaterally at 2- or 4-cell stage with either 0.5 ng *Znf703* (**A**–**F’**) or control *mCherry*. (**A**–**C**) *Znf703* mRNA reduces the lateral and anterior expression of *Sox10*, *Twist*, and *Foxd3* in stage 19 embryos (shown in anterior view). Injected side is to the right of the dotted line, and is indicated by the magenta *β-gal* lineage tracer. (**D’**–**F’**) *Znf703* mRNA inhibits *Sox10* patterning, *Twist*, and *Tfap2a* in stage 27 embryos (shown in lateral view). (**D**–**F**) Uninjected side of the same embryo. Fractions represent the portion of embryos displaying the phenotype.

Recent data has shown that modulation of *Znf703* activity affects neural crest markers *Sox10* and *Snai2*^6^. When *Znf703* is overexpressed, we observed knockdown of early neural crest markers *Foxd3* and *Tfap2a* (Fig. S2). At later stages, *Sox10, Twist*, and *Foxd3* expression domains do not separate into distinct migratory streams, compared to the uninjected side (Fig. 4A–C). Rather, the cells marked by *Sox10, Twist*, and *Foxd3* are aggregated together with no discernible pattern. By tailbud stage, this effect is further manifested in the lack of ventral migration of crest markers into the epibranchial domain (Fig. 4D’-F’). Manipulation of the FKPY (putative Groucho) domain of *Znf703* did not affect the overexpression phenotype of *Znf703* on the markers we tested (Fig. S3).

**Figure 5:**
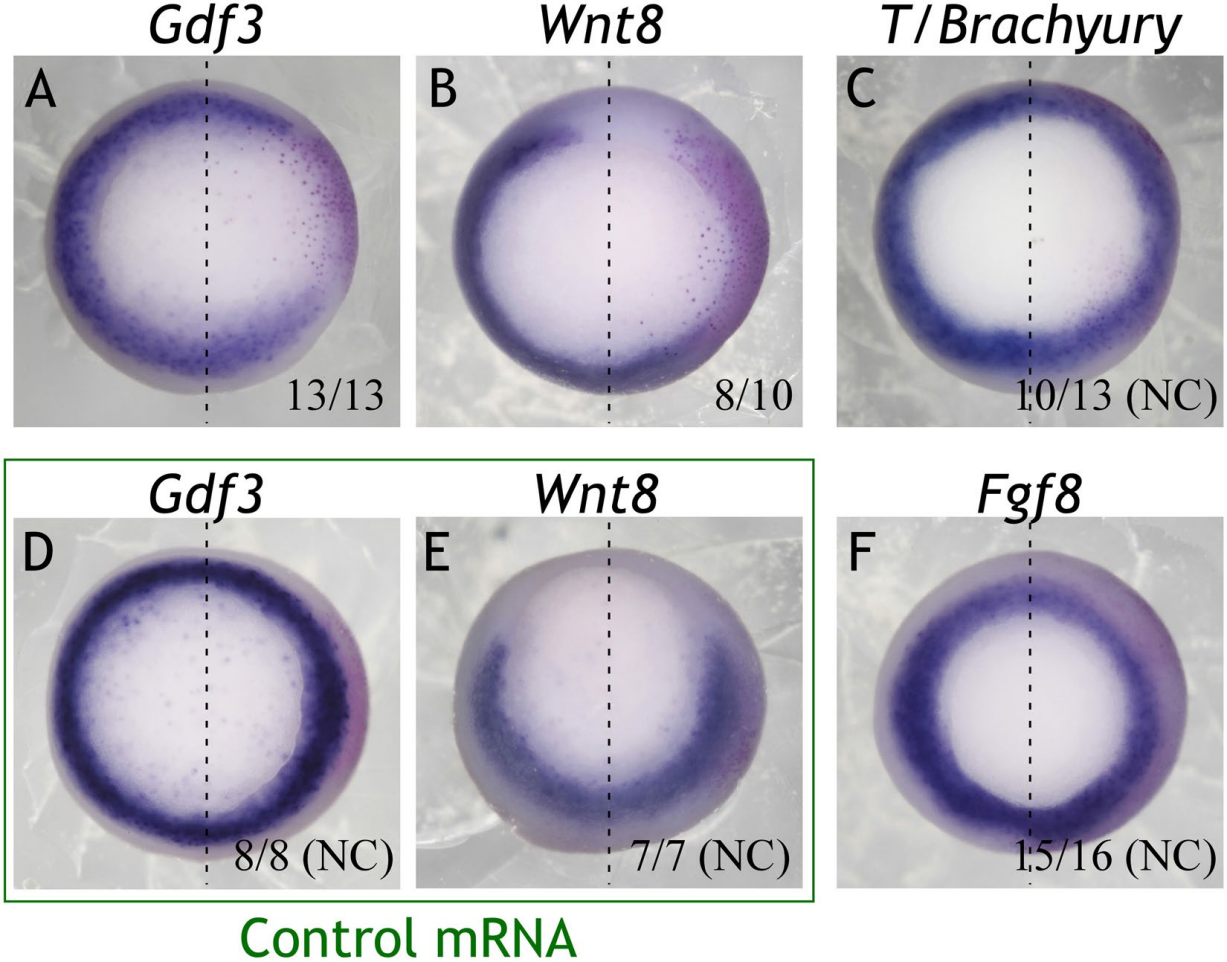
Overexpression of *Znf703* mRNA reduces expression of *Gdf3* and *Wnt8*, but not *T* or *Fgf8*. Embryos were injected unilaterally at 2- or 4-cell stage with either 0.5 ng *Znf703* or control *mCherry* mRNA. Injected side is to the right of the dotted line, and is indicated by the magenta *ß-gal* lineage tracer. (**A**–**C**,**F**) *Znf703* mRNA causes loss of *Gdf3*, knockdown of *Wnt8*, and weak knockdown or no change of T and Fgf8. Embryos are shown at stage 10.5/11 in vegetal view. (**D**,**E**) Control mRNA did not have any effect on *Gdf3* or *Wnt8*. Fractions represent the portion of embryos displaying the phenotype. NC = No change.

**Figure 6:**
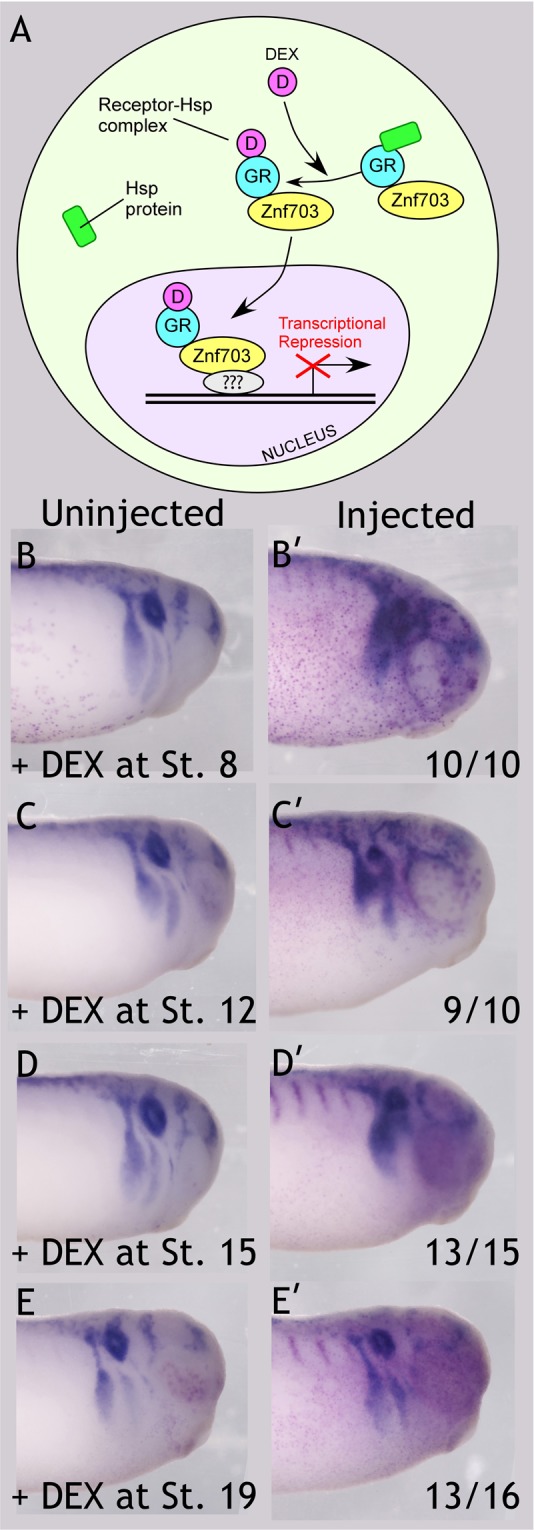
*Znf703* impairs *Sox10* expression at tailbud stage, when induced after stage 12 up until stage 19. (**A**) Schematic of ligand-inducible transcriptional repression by hGR-*Znf703*. In the absence of dexamethasone (DEX), hGR-ZNF703 is tethered in the cytoplasm by HSP90. In the presence of DEX, hGR-ZNF703 is free to enter the nucleus. Question marks indicate that *Znf703* is not thought to bind DNA directly. (**B**–**E’**) Embryos were injected unilaterally with 0.2 ng hGR-*Znf703* mRNA at 2- or 4-cell stage, then treated with 5 µM DEX or 0.05% DMSO vehicle at the stages indicated. Injected side is indicated by the magenta *β-gal* mRNA lineage tracer. *Znf703* blurs the migratory streams of *Sox10* expression when *Znf703* is induced after gastrulation and prior to stage 19. Little effect on *Sox10* is observed when embryos are treated with DEX at stage 19. DMSO treated embryos are pictured in Fig. S4. All embryos are shown in lateral view with anterior on the right, at stage 27. Fractions represent the portion of embryos displaying the phenotype in one time-course experiment from the same clutch of embryos. This experiment was repeated an additional time, and very similar results were obtained.

Manipulation of *Znf703* yields nearly identical phenotypes in loss-of-function and gain-of-function experiments^6^. Such outcomes can often be ascribed to the manipulation of a gene that has a different function early versus late in development, and disrupting both functions can confound interpretation of results^28^. *Znf703* is such a gene since it is expressed in early mesoderm (Fig. 2A). Mis/overexpression of *Znf703* yields selective loss of mesodermal markers *Wnt8* and *Gdf3* (Fig. 5A,B) without affecting *T/Brachyury* or *Fgf8* (Fig. 5C,F); control mRNA (*mCherry*) did not alter *Wnt8* or *Gdf3* expression (Fig. 5D,E). There is ample evidence that early blastula and gastrula stage events (mesoderm formation, hypoblast signaling, pluripotency retention, etc.) contribute to neural crest and placode development^29–33^. We hypothesized that early loss of *Wnt8* and/or *Gdf3* could contribute to the effect of *Znf703* on neural crest patterning. To test this hypothesis, we designed dexamethasone (DEX)-inducible^34^ hGR-*Znf703* constructs to separate the effects of *Znf703* at gastrula, neurula and tailbud stages (Fig. 6A). A preliminary titration revealed the dose of mRNA (0.2 ng) needed to avoid saturation of the heat-shock protein which tethers hGR-*Znf703* outside of the nucleus until DEX binding. This is the dose at which we observed no effect on *Sox10* expression in vehicle (DMSO) treated embryos (Fig. S4).

When *Znf703* expression was induced prior to gastrulation (stage 8) we observed loss of *Wnt8* and *Gdf3* (Fig. S5), confirming that the hGR-Znf703 construct yields the same results as wildtype *Znf703*. When *Znf703* is induced after gastrulation (stage 12 or 15), neural crest gene expression is not noticeably altered at stage 18 (Fig. S6). However, by stage 27, we found that clarity in the segregation of migratory streams is lacking, and the otocyst is compromised (Fig. 6C–D’). This effect on *Sox10* was nearly identical to *Znf703* mis/overexpression induced before gastrulation (cf Fig. 6B,B’) or non-inducible *Znf703* mis/overexpression (Fig. 4D,D’). We observe a slight improvement in the size and posterior-lateral positioning of the otocyst when DEX is provided at stage 15, but epibranchial and lateral line patterning remain perturbed at tailbud stage. From this, we conclude that the early loss of *Gdf3* and *Wnt8* (Figs 5A,B and S5) by *Znf703* mis/overexpression does not significantly impact *Sox10* expression later. Hence, *Znf703* has a different and separable function in mesoderm versus neural crest development. Finally, treatment with DEX at stage 19 showed minimal effect on *Sox10* expression (Fig. 6E,E’).

### Znf703 promotes anterior SOX2 expression at the expense of otic development

**Figure 7:**
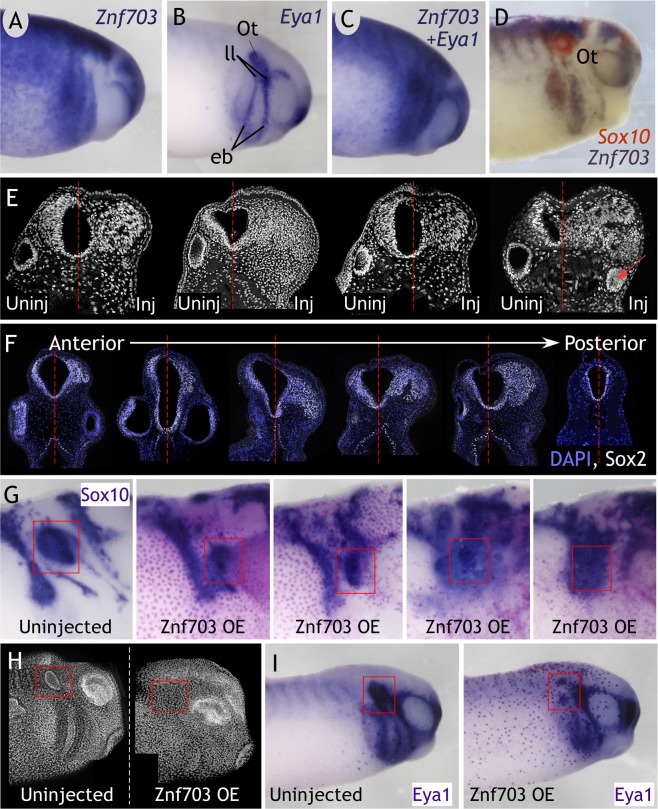
*Znf703* is normally absent from the otocyst. *Znf703* overexpression causes aberrant *SOX2* expression and disappearance of otocyst. (**A**–**D**) *Znf703* is absent from the otocyst as marked by *Eya1* and *Sox10*, and lateral line placode as marked by *Eya1*, shown in lateral view at stage 27. Ot = otocyst; ll = lateral line; eb = epibranchial placodes. Embryos were injected unilaterally at the 2- or 4-cell stage with 0.5 ng *Znf703* mRNA (**E**,**G**–**I**) or 0.2 ng hGR-*Znf703* mRNA and treated with DEX at stage 12 (**F**). (**E**,**F**) Maximum intensity projections of Dapi nuclear stain and/or SOX2 from confocal images of transverse sections (expansion of SOX2 observed in 8/8 embryos sectioned). Otocysts were not found on the injected side in 60% of embryos. In the remaining 40% of embryos, the otocyst was reduced in size (red arrow). (**G**) *Sox10* expression marks otocysts that are significantly reduced in size and positioned more rostral/ventral in the head compared to control (Stage 27 embryo in lateral view). (**H**) Maximum intensity projection of Dapi (false-colored in white) from confocal images through a laterally-mounted stage 27 embryo. (**I**) *Eya1* expression is reduced, particularly in the otic vesicle on the injected side (Stage 27 embryo in lateral view) (15/15 embryos). Red boxes in H and I highlight the otocyst on the uninjected side and its absence on the injected side.

In tailbud embryos, *Znf703* is strikingly absent from the otocyst, epibranchial placode, and lateral line, where *Eya1* is normally expressed (Fig. 7A–C). *Eya1* is a weak probe for double WISH, therefore, we stained with the stronger *Sox10* probe along with *Znf703* to confirm that *Znf703* is completely absent from the otocyst (Fig. 7D). Upon sectioning tailbud-stage embryos microinjected with *Znf703* after gastrulation, we consistently observed the absence of an otocyst on the injected side in transverse sections through the head (Fig. 7E,F). Similarly, in laterally-mounted whole embryos, confocal imaging through the surface towards the midline did not reveal an otocyst on the injected side (Fig. 7H), and otic markers *Eya1 and Sox10* were significantly reduced (Fig. 7G,I). Concurrently, we observed a massive overgrowth of cells in the anterior neural tube (Fig. 7E), which we determined to be SOX2-positive (Fig. 7F). This proliferative effect is also observed when *Znf703* is induced after gastrulation, and is only seen in the anterior domain: the degree of SOX2 expansion diminished in sections taken posterior to the otocyst (Fig. 7F). The fate of these SOX2-expressing cells remains unclear, because *Sox2* is widely expressed in development. Perhaps one of the more well-known developmental processes of *Sox2* is in the neurogenic lineage, but we found no increase in primary neurons when *Znf703* is overexpressed (Fig. S7). Alternatively, increased *Sox2* expression could be promoting cell proliferation without driving a specific cell fate.

## Discussion

### Znf703 is an RAR-inducible transcriptional repressor

*Znf703* is an intriguing potential effector of cross-talk between RA, *Wnt*, *Noggin*, and *Nodal* signaling. *Znf703* has been associated with both *Nodal* and *Noggin*^6,35^ and is a direct target of *Wnt* pathways^8,11^. *Znf703* is induced by RA in P19 embryonal carcinoma cells and in zebrafish^14,36^ and was also identified by microarray to be RA-inducible the E8.5 mouse^37^. The data we have presented further affirms *Znf703* as an RA responsive gene *in vivo*. Whether *Znf703* is also a direct target of RAR is an open question. We found no discernable RAR binding site within 20 kilobases upstream or downstream of *Znf703*, or within the gene itself. Existing ChIP data in mouse *in vitro* studies do not identify *Znf703* as a direct target^38–40^. This does not prove that the interaction between RAR and *Znf703* is indirect, but rather indicates that additional, *in vivo*, ChIP analysis will be required to conclusively establish whether Znf703 is a direct or indirect target of RA. Furthermore, RAR occupancy on DNA does not necessarily equal transcriptional activation since RARs can act as repressors in the absence of RA^41,42^.

When *Znf703* was originally characterized, it was hypothesized that the FKPY domain was recruiting the transcriptional corepressor *Groucho* based on its similarity to *Brinker* and *Elbow* FKPY^24,25^ and *Hucklebein* FRPW^26^, domains that are required for *Groucho* interaction. Murine *Zfp703* and *Zfp503* have been named *Zeppo1* and *Zeppo2*^9,43^, that are undoubtedly referencing the relationship to Groucho and Zeppo Marx. However, deletion analysis in zebrafish showed that there was no effect on the Groucho interaction when the N-terminus and FKPY of *Znf703/Zeppo1* were removed^5^. In addition, the FKPY motif in *Brinker* did not affect the ability of a stripe2-Brinker transgene to repress *dpp*^25^. Similarly, we demonstrated that mutating the FKPY domain did not affect transcriptional repression, nor did it exacerbate or weaken phenotypes associated with wild type *Znf703* expression on mesoderm or neural crest markers. Thus, we infer that transcriptional repression by *Xenopus Znf703* requires the *Buttonhead* domain. Future studies will investigate how the *Buttonhead* domain of *Znf703* mediates transcriptional repression during embryonic development and whether a combination of domains is required for maximal repression, *in vivo*.

### Znf703 overexpression hinders neural crest development after gastrulation

Misexpressing or overexpressing a transcriptional repressor from early stages in development can often result in confounding phenotypic effects^28^. Gain- and loss-of-function analysis of *Znf703* both resulted in loss of neural crest genes *Snai2* and *Sox10* ^6^. We showed that *Znf703* inhibits expression of mesodermal markers *Gdf3* and *Wnt8* at gastrula stage and disrupts neural crest migration and patterning in the neurula and tailbud stage embryo. The dorsolateral marginal zone in *Xenopus*, where *Znf703* is co-expressed with *Wnt8* and *Gdf3*, is thought to be responsible for neural crest induction^29–31^. Furthermore, if loss of *Wnt8* in *Znf703*-injected embryos is indicative of Wnt levels in the early gastrula, then neural crest competence would be compromised^32^. Therefore, it is not inconceivable that early deficiency in *Wnt8* and *Gdf3* expression at gastrula stage would have direct consequences on neural crest later.

To test this possibility, we designed hormone-inducible *Znf703* expression constructs. We found that *Znf703* is still fully capable of perturbing neural crest and placode patterning at tailbud stage when induced after stage 12, thus demonstrating that *Znf703* has a role in early mesoderm development that is separable from its effects on neural crest and placode. A proline- and tyrosine-rich domain in the C-terminus ensures *Znf703* and *Znf503* nuclear localization^4,43,44^; however, with only one zinc finger present, it is assumed that these proteins lack the ability to bind DNA directly^45^. *Znf703* and *Znf503* are related to the SP family of proteins which can function as both co-activators and co-repressors^46^. Thus, *Znf703* likely behaves as a transcriptional cofactor, potentially with multiple binding partners depending on the cellular context. Based on our results, it is plausible that *Znf703* possesses a different interactome in mesoderm versus neural crest, which is an interesting area of future study.

Our hormone-inducible *Znf703* experiments clearly resolve early (gastrula) versus late (neurula) events, but we can also propose a role for *Znf703* in induction/specification, delamination and migration of the neural crest. When Dex is administered at stage 15, the *Znf703* protein is likely to translocate into the nucleus by around stage 18 (two hours later^47^), when neural crest cells are beginning to segregate and migrate, but induction/specification has already transpired^48^. At stage 18, we cannot detect any changes in neural crest patterning when *Znf703* is induced at stage 12 or stage 15. However, by stage 27, we observed significant loss of defined *Sox10* migratory streams. These data are consistent with a model in which *Znf703* is primarily affecting delamination/migration, which agrees with its known role in epithelial-mesenchymal transitions (EMTs) and regulation of *E-cadherin*^9,43^. Induction of *Znf703* beyond stage 19 was no longer detrimental to neural crest patterning. Nevertheless, we did not evaluate bone, cartilage or pigment in tadpoles to completely rule out the role of *Znf703* in neural crest differentiation.

### Znf703-induced SOX2 expansion at the neural plate border

Our results and those of Hong, 2017 have established that *Znf703* is expressed in the neural plate border. The neural plate border is a small region where progenitors of multiple different lineages mingle and signal dynamically over very small distances^1^. Recently, this was shown at the single cell, molecular level where early neural plate stages show significant overlap of transcription factor expression that later become restricted to separate lineages^2^. Misexpression of *Znf703* expands *Sox2* mRNA expression at the open neural plate stage 14/15, and this expansion seemed more pronounced in the anterior^6^. In agreement with these data, we showed that *Znf703* mis/overexpression causes prodigious expansion of the SOX2 protein domain at tailbud stage, observable only in the head.

A preponderance of ectopic SOX2+ expressing cells found in the anterior neural plate of *Znf703*-overexpressing embryos will undoubtedly upset the balance of intricate signaling and lineage decisions occurring at the neural plate border. Misexpression of *Sox2* in quail inhibits *Slug* expression and neural crest migration^49^. *Sox3* gain-of-function delays neural crest induction, reduces migration, and disrupts branchial cartilage development, while promoting the neural progenitor fate^50^. The precedent for *Sox2/3* overexpression altering the boundary between neural and non-neural ectoderm supports the argument that the SOX2 expansion we observe is causal to the loss of *Foxd3* and *Tfap2* in the early neurula. Nevertheless, the consequence of copious numbers of SOX2+ cells, induced by *Znf703*, is not easily interpretable given that the SoxB1 family is notoriously perplexing with respect to its spatial and temporal expression and regulation of proliferation/pluripotency, cell lineage, and differentiation. It is unclear whether extra SOX2+ cells are simply indicative of proliferation, without an associated lineage, or if SOX2 is preferentially driving a specific cell fate, at the expense of another. Inducible *Eya1* overexpression phenocopies our results on *Sox2*, including the failure of the otic vesicle to form Fig. 7 ^51^. These authors concluded that ectopic SoxB1 stabilized a placodal, neurogenic progenitor fate, at the expense of differentiation^51^. Similarly, we found that *Znf703* did not increase *N-tubulin* expression after gastrulation, therefore, SOX2-expressing cells might commit cells towards a neurogenic lineage, but cannot drive differentiation without additional signals. As a result, the SOX2-expressing cells might later die, since they are unable to differentiate at the correct time and place in the embryo. Alternatively, the ectopic SOX2+ cells contribute to neural crest and epidermal derivatives as found recently in chicken by Roellig and colleagues, 2017^2^. Deciphering the identity of *Znf703*-induced SOX2+ cells, is an interesting area of future study.

### Znf703 and otic development

Our group has a long-standing interest in RA and placode development. We previously found that RA is essential for establishing the posterior-lateral boundary of the preplacodal ectoderm^18^. *Znf703* (orthologous to the invertebrate *no ocelli*) has been linked to sensorineural development throughout evolution^52,53^. *Nlz* morphant zebrafish fail to close the optic fissure in the ventral region of the developing eye^54^. *Znf503* is a negative regulator of *Gata3*^55^ which is expressed in the preplacodal ectoderm^56^ and is a critical factor in specifying the prosensory domain of the otocyst^57,58^. *Znf703* is also cistronic to *Fgfr1*, important for maintaining *Sox2*+ progenitors in the Organ of Corti^59^.

In this current study, we noted that *Znf703* is almost an inverted image of *Eya1*, as revealed by double WISH. Transverse sections through *Znf703*-injected embryos at the r5/r6 level showed otocyst absence or reduction in size, as well as changes in its posterior-lateral position in the head, and subsequent loss of *Eya1* and *Sox10* otic expression. The implication is that local *Znf703* must be specifically cleared or down-regulated in the developing otocyst, otherwise *Znf703* would inappropriately repress genes required for ear development. The developing embryo is exquisitely sensitive to RA concentration; therefore, local retinoid levels in the otocyst must be tightly regulated (reviewed in^60^). Local boundaries of RA during development are often established by opposing Fgf signals^61^. One possible scenario is that RA induces *Znf703* to inhibit *Gata3*, whose primary target is *Fgf10*^62^, thus RA would inhibit FGF signaling via *Znf703*. A second possibility concerns RA-*Wnt* interactions. *Wnt* signaling is required for specifying the otic fate, and when *Wnt* is inhibited, the placode territory instead becomes epidermal or epibranchial^63,64^. Others have established *Znf703* as a Wnt inhibitor; therefore, *Znf703* could regulate the fate decision and/or boundary delineation of the epidermal/epibranchial (low *Wnt*) versus otic (high *Wnt*) lineages by modulating Wnt signaling.

## Conclusion

*Znf703* is an important point of crosstalk among RAR, WNT, and TGFβ (among other) signaling pathways, and exhibits a complex expression pattern early in the circumblastoporal ring and neural plate border, and later in crest, placode, hindbrain, and somites. In this paper, we focused on addressing the role of *Znf703* in neural crest and otic development, but *Znf703* is likely to affect other lineages at the neural plate border such as anterior placode (e.g., lens) and mesodermal derivatives (somites). *Znf703* is poised to interpret local RA levels while, simultaneously, modulating Wnt signaling. Therefore, *Znf703* is anticipated to be an important ingredient in many embryonic processes where Wnt-RA crosstalk is required. *Znf703* is unlikely to bind DNA directly, and therefore, *Znf703* may potentially be promiscuous in its interaction with other transcription factors. Detailed biochemical analysis will be required to understand how *Znf703* functions, which co-repressor(s) interacts with *Znf703*, and which exact domain, or combination of domains are responsible for transcriptional repression. Finally, the translational implications of RA-regulation of *Znf703* are intriguing because RA is known to improve outcomes in a limited number of cancers^65^, but *Znf703* acts an oncogene to promote adult luminal B breast cancer in humans^35^.

## Supplementary information

Supplemental Figures and Tables
