# Supplementary material for: Znf703 is a novel RA target in the neural plate border
Source: Sci Rep. 2019 Jun 4;9:8275. doi: 10.1038/s41598-019-44722-1 (PMC6547707; doi:10.1038/s41598-019-44722-1)
Supplement: Supplementary file 1 — Supplemental Figures and Tables [file 41598_2019_44722_MOESM1_ESM.pdf]

Supplementary Information

Znf703 is a novel RA target in the neural plate border

Amanda Janesick, Weiyi Tang, Kristen Ampig, Bruce Blumberg

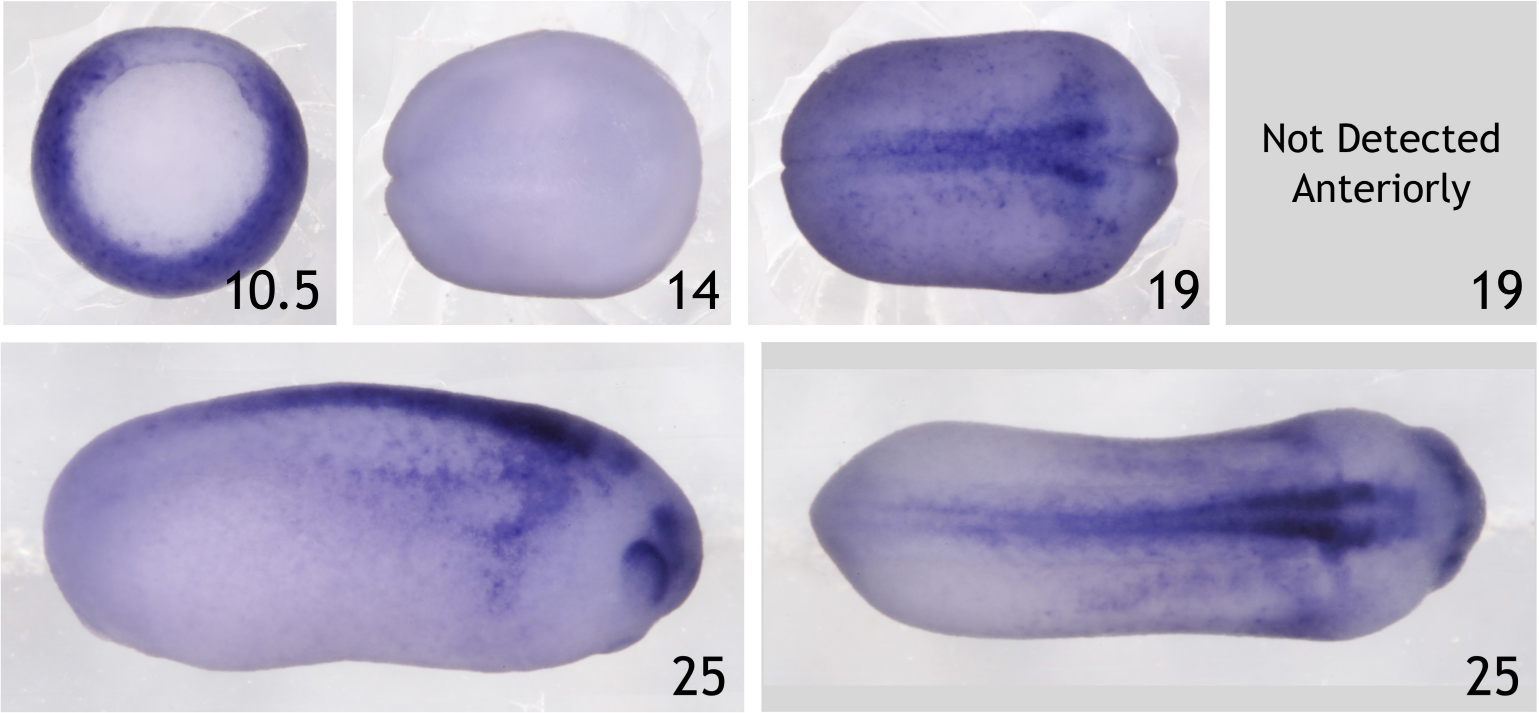

Supplemental Figure S1. Expression of *Znf503* across developmental time. Whole mount in situ hybridization of *Znf503* expression at Nieuwkoop and Faber developmental stages 10.5 (vegetal view, dorsal at the top), 14 (dorsal view, anterior on the left), 19 (dorsal and anterior views), and 25 (lateral and dorsal views, anterior on the right).

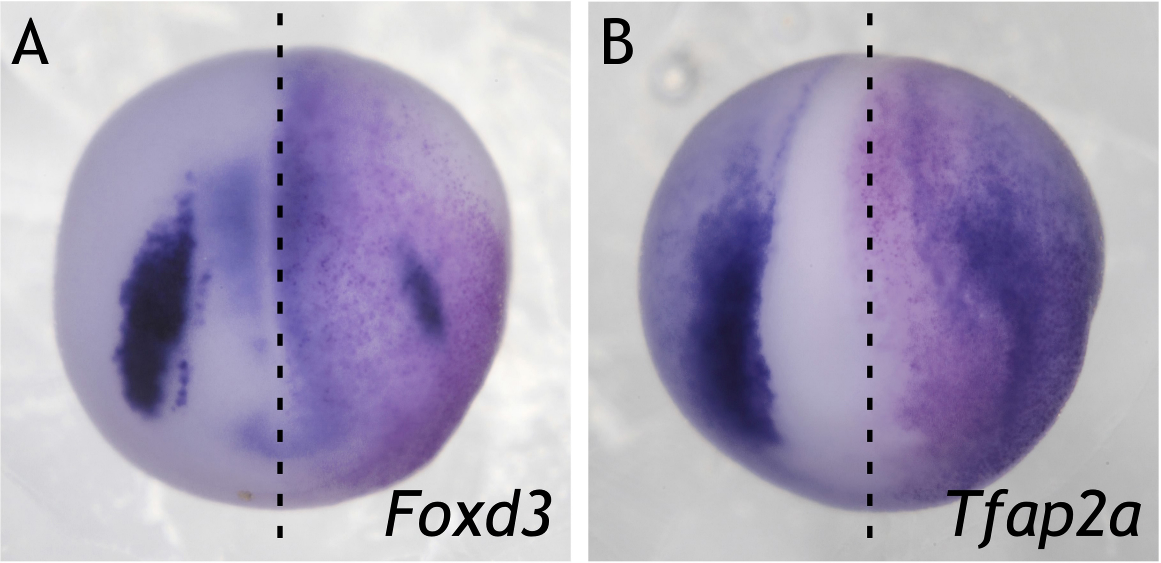

Supplemental Figure S2. Overexpression of *Znf703* mRNA inhibits early neural crest markers. Embryos were injected unilaterally at 2- or 4-cell stage with 0.5 ng *Znf703* mRNA. Injected side is to the right of the dotted line, indicated by magenta *β-gal* lineage tracer. Embryos are shown at stage 14 in anterior view. *Znf703* mRNA knocks down expression of **(A)** *Foxd3* and **(B)** *Tfap2a*.

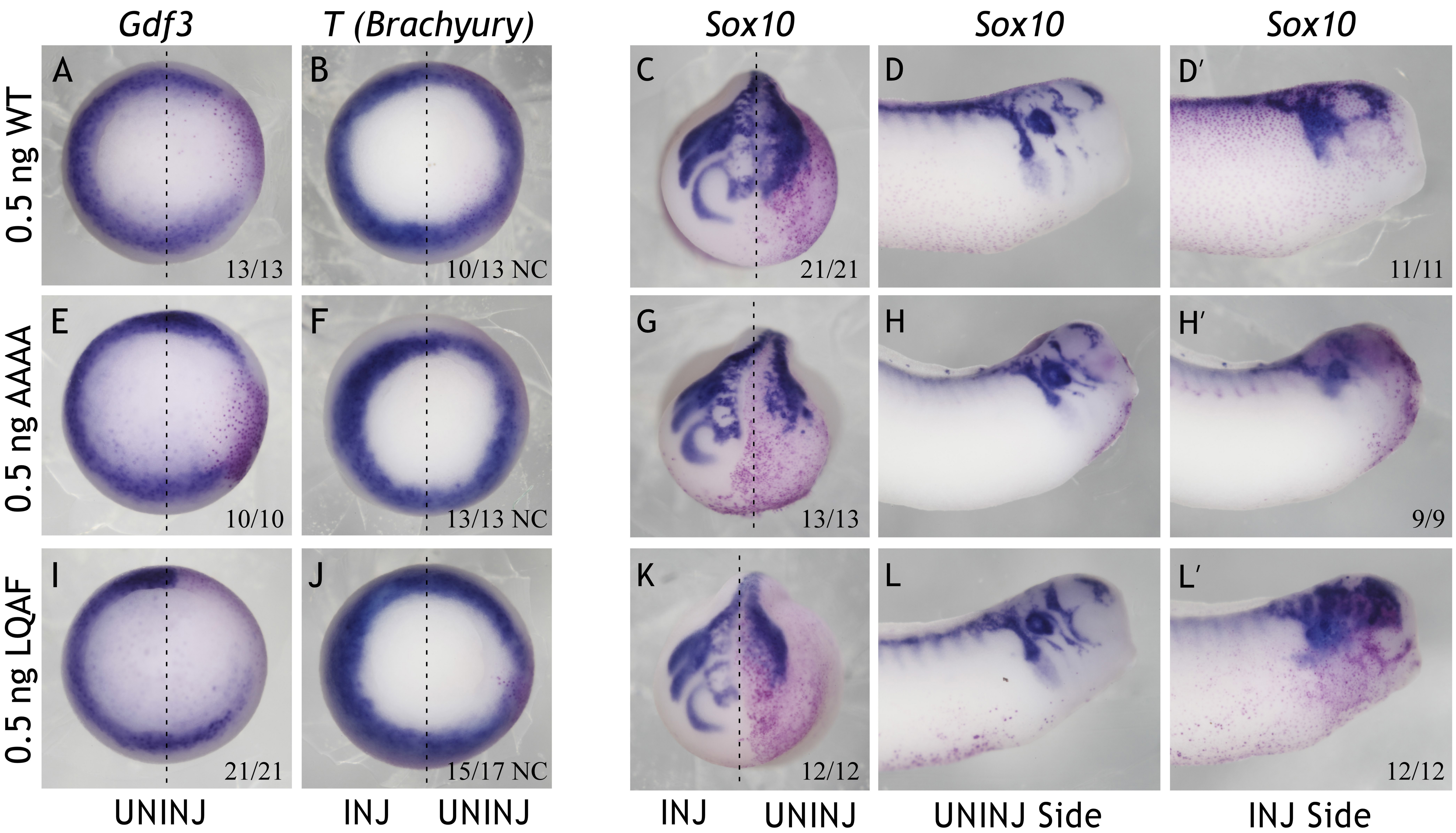

Supplemental Figure S3. Mutation of the FKPY domain does not alter the *Znf703* overexpression phenotype on *Gdf3*, *Wnt8* or *Sox10*. Embryos were injected unilaterally at 2- or 4-cell stage with 0.5 ng *Znf703* wildtype or mutant (FKPY→AAAA or FKPY→LQAF) mRNA. **(A, B, E, F, I, J)** Stage 10.5/11 embryos shown in vegetal view with injected side to the right of the dotted line, indicated by the magenta *β-gal* lineage tracer (LT). *Znf703* mRNA WT or mutant caused knockout of *Gdf3* and no change or very weak knockdown of *T.* **(C, G, K)** Stage 19 embryos in anterior view with injected side to the right of the dotted line, indicated by the LT. *Znf703* WT or mutant mRNA reduces the lateral and anterior expression of *Sox10*. (**D, H, L**) Stage 27 embryos in lateral view showing the uninjected side or injected side (**D′, H′, L′**). *Znf703* mRNA inhibits the ventral migration, and disrupts patterning of *Sox10*. Fractions represent the portion of embryos displaying the phenotype. NC = no change.

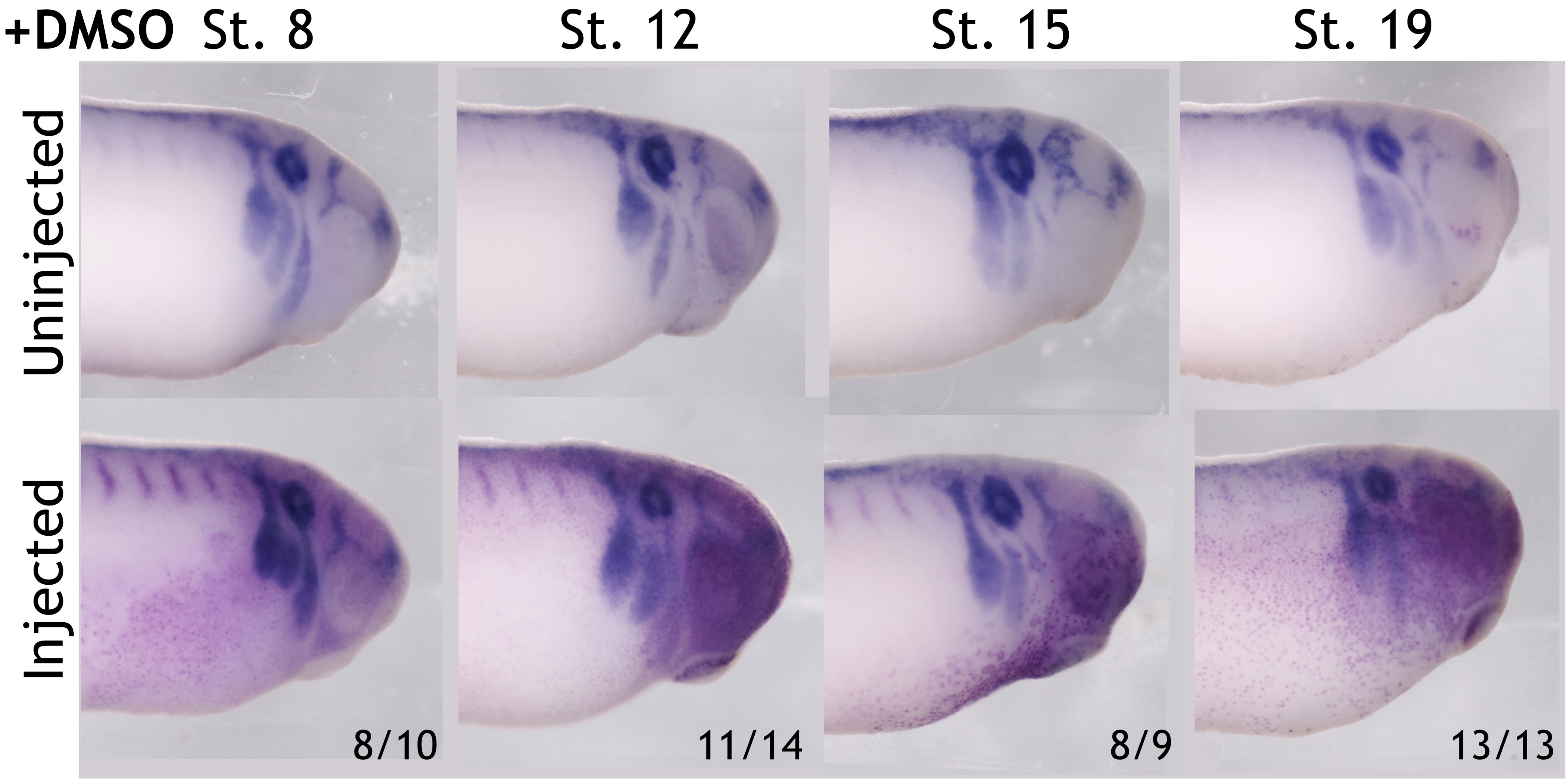

Supplemental Figure S4. hGR-*Znf703* has no effect on *Sox10* expression in the absence of DEX. Embryos were injected unilaterally with 0.2 ng hGR-*Znf703* mRNA at 2- or 4-cell stage, then treated with 5 μM DEX or 0.05% DMSO vehicle at the stages indicated. Injected side is indicated by the magenta *ß-gal* mRNA lineage tracer. DEX treated embryos are pictured in **Fig. 6**. hGR-*Znf703* overexpression in the presence of DMSO control vehicle shows little or no effect on *Sox10* expression at all stages tested. All embryos are shown in lateral view with anterior on the right, at stage 27. Fractions represent the scoring of embryos displaying the phenotype of no change compared to the uninjected side.

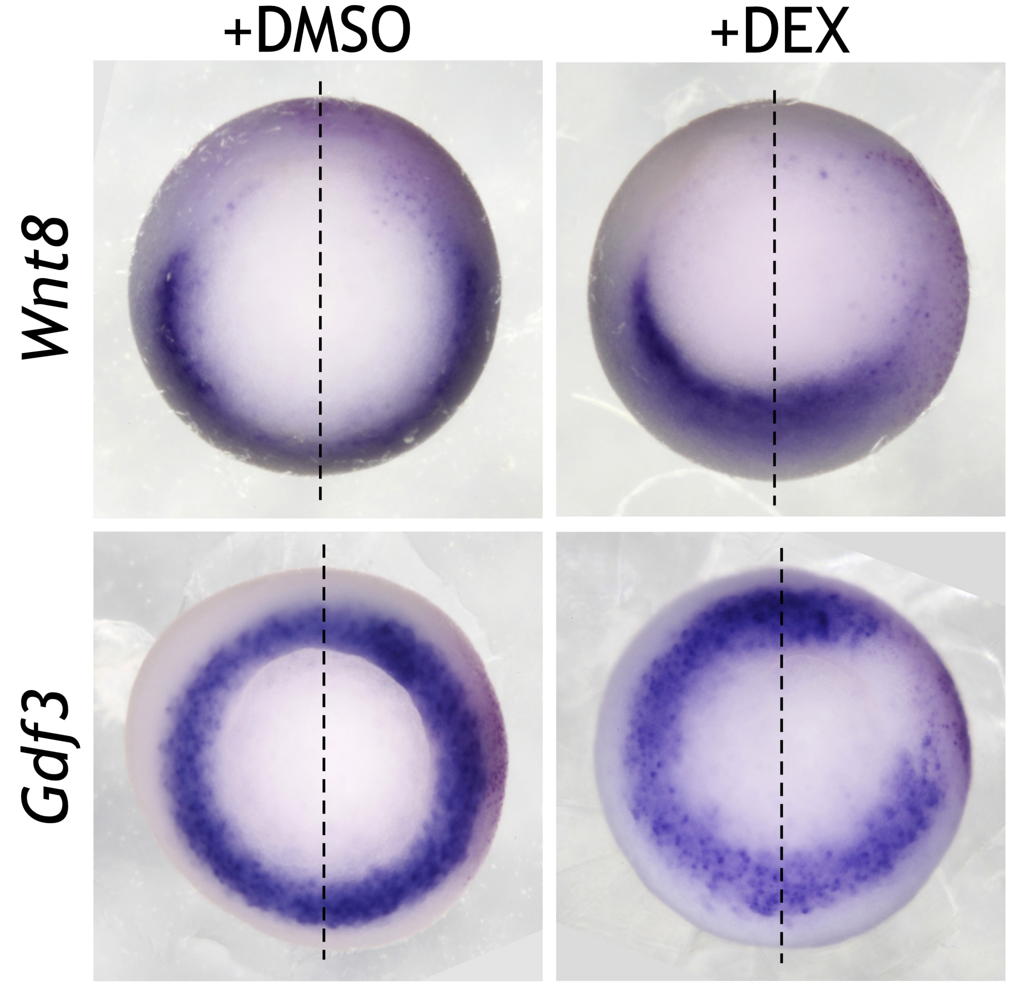

Supplemental Figure S5. hGR-*Znf703* yields the same result as wildtype Znf703 on mesodermal markers. Embryos were injected unilaterally with 0.2 ng hGR-*Znf703* mRNA at 2- or 4-cell stage, then treated with 5 μM DEX or 0.05% DMSO vehicle at stage 8. Injected side is indicated by the magenta *ß-gal* mRNA lineage tracer. Stage 11 embryos are shown in vegetal view with the dorsal lip at the top.

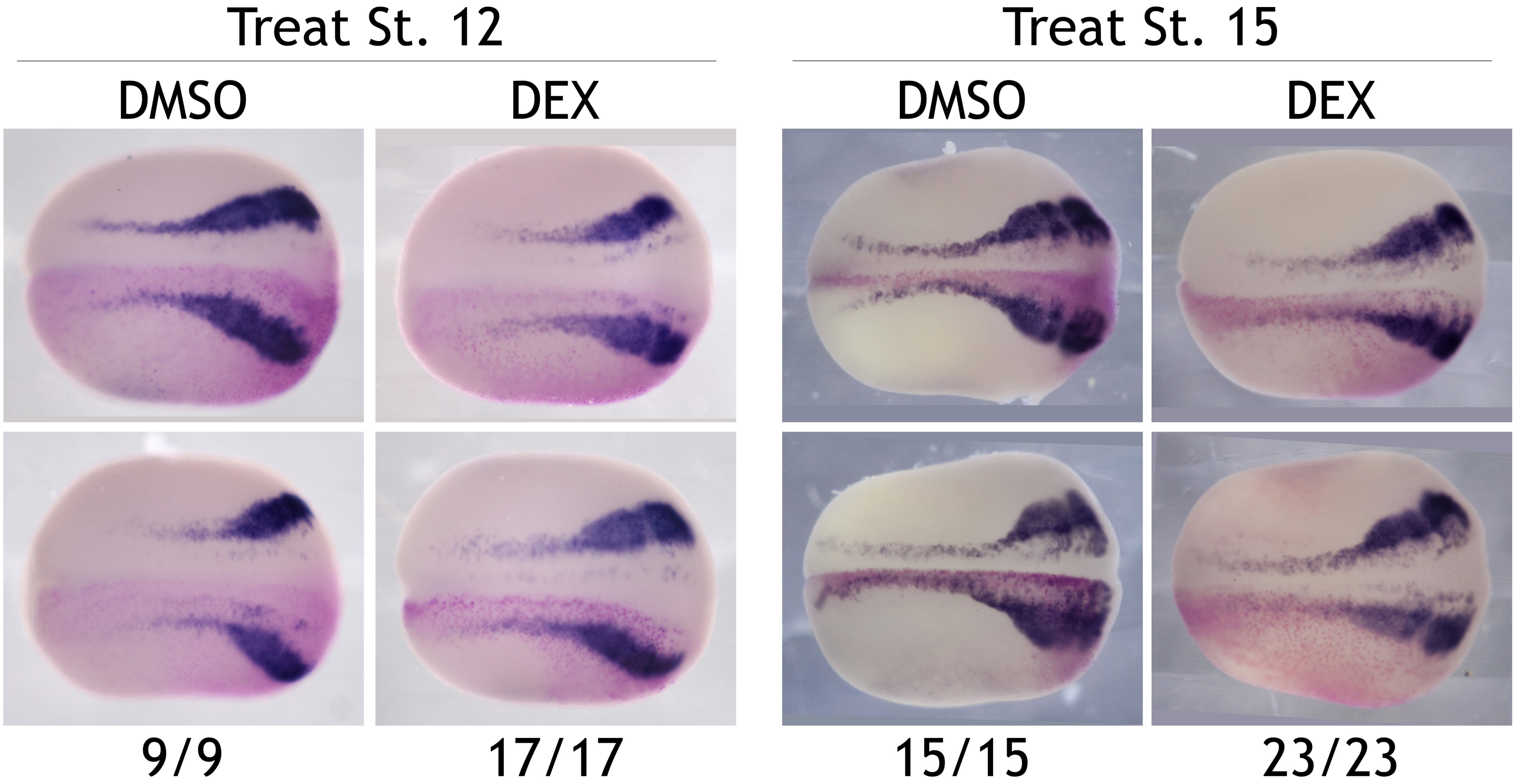

Supplemental Figure S6. hGR-*Znf703* induced at stage 12 or 15 has no effect on *Slug* expression at stage 18/19. Embryos were injected unilaterally with 0.2 ng hGR-*Znf703* mRNA at 2- or 4-cell stage, then treated with 5 μM DEX or 0.05% DMSO vehicle at the stages 12 or 15. Injected side is indicated by the magenta *β-gal* mRNA lineage tracer. hGR-*Znf703* overexpression in the presence of DMSO vehicle or DEX shows no effect on *Slug* expression at stage 18/19. All embryos are shown in dorsal view with anterior on the right. Fractions represent the scoring of embryos displaying the phenotype of no change compared to the uninjected side.

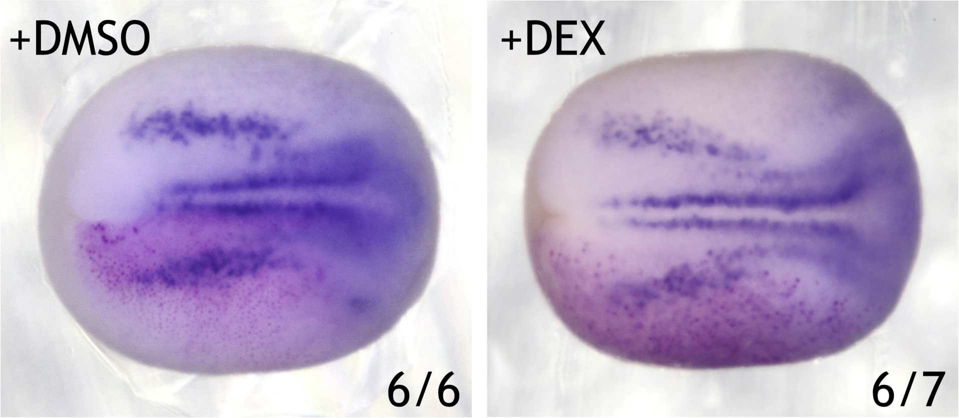

Supplemental Figure S7. hGR-*Znf703* does not increase neuronal differentiation. Embryos were injected unilaterally with 0.2 ng hGR-*Znf703* mRNA at 2- or 4-cell stage, then treated with 5 μM DEX or 0.05% DMSO vehicle at stage 12. Injected side is indicated by the magenta *β-gal* mRNA lineage tracer. All embryos are shown in dorsal view at stage 14 with anterior on the right.

Supplemental Table S1 (Probe Design)

Probes with T7 Adapters

Primer	Sequence (5'→3')
Znf703_Probe_For	AGCTGAATTCTGTGACCTCCAG
Znf703_Probe_Rev	taatacgactcactatagggCATAAAGCCGTAGGTGTACAAGG
Znf503_Probe_For	CCACTGGGTTCTGGAAGTCG
Znf503_Probe_Rev	taatacgactcactatagggTTTATAGGGTGACACAGGTGC
Sox10_Probe_For	GATGACCAAAGCTTGTCCGA
Sox10_Probe_Rev	TAATACGACTCACTATAGGGgctgttcccaatgtgtagg
Twist1_Probe_For	GACAGTCTGAGTAACAGCGAGGA
Twist1_Probe_Rev	taatacgactcactatagggTCCACACGGAGAAGGCATAGC
Tfap2a_Probe_For	CCCAGTCTCAAGATCCCTACTCC
Tfap2a_Probe_Rev	taatacgactcactatagggCCGATTGACAAATTCAGCCGC
Foxd3_Probe_For	GGACAAGGACAGTGAGTGCG
Foxd3_Probe_Rev	taatacgactcactatagggTCTATGCTGAAGGAGGGTCGG
Snai2_Probe_For	GTCAAGAAACACTTCAACTCGGC
Snai2_Probe_Rev	taatacgactcactatagggACAGCAACCAGATTCCTCATGCT
Eya1_Probe_For	GTTCGCCGTATCCTTCACAT
Eya1_Probe_Rev	taatacgactcactatagggTGAATGCTTTTTGGCTCCTT

Chicken_Znf703_Probe_For	CCTTCCAGCATCGGTTACCAC
Chicken_Znf703_Probe_Rev	taatacgactcactatagggTGGCAAACCTCTTATCGCAG

Supplemental Table S2 (RT-QPCR)

Primer	Sequence (5'→3')
Znf703_QPCR_For	GCTGATCAACATGCTGACGG
Znf703_QPCR_Rev	CGGCTTCCCTATCTGTGAGC
Znf503_QPCR_For	CCCGAATACCTTCAACCTCTGC
Znf503_QPCR_Rev	GTCTGGCTTCCCGATCTGTG

Supplemental Table S3 (Two-Fragment PCR)

Primer	Sequence (5'→3')
pCDG1_Znf703.L_For (A)	CAGATACCATGGACTGTTCTCCCCCTGGATCTAGC
pCDG1_Znf703.L_LQAF (B)	CCCTTCGAAAATGCTTGCAAGCTAGACTTATCCTCCAAAGGAGA
pCDG1_Znf703.L_AAAA (B)	CCCTTCGAAGCTGCAGCTGCGCTAGACTTATCCTCCAAAGGAGA
pCDG1_Znf703.L_LQAF (C)	CTAGCTTGCAAGCATTTTCGAAGGGTGGGGAGACCAG
pCDG1_Znf703.L_AAAA (C)	CTAGCGCAGCTGCAGCTTCGAAGGGTGGGGAGACCAG
pCDG1_Znf703.L_Rev (D)	ACTAGTGGATCCCTATTACTGGTATCCTAGCGCTGAAG

Supplemental Table S4 (Gal-Znf703)

Primer	Sequence (5'→3')
pCMX-GAL_Zeppo1_For	TCGCCGGAATTCATGGACTGTTCTCCCCCTGGATC
pCMX-GAL_Zeppo1_(ZFD)_For	TCGCCGGAATTCTCTGGTCACCCCTTGTACACCTAC
pCMX-GAL_Zeppo1_(Btd)_For	TCGCCGGAATTCTCCAGCCCTCTGACTGGTGC
pCMX-GAL_Zeppo1_Rev	TGGCCAGGATCCCTATTACTGGTATCCTAGCGCTGAA
